# Age-related changes in auditory perception. Hearing loss in the elderly: aging ear or aging brain?

**DOI:** 10.1007/s40520-023-02570-0

**Published:** 2023-10-13

**Authors:** Davide Brotto, Francesco Benvegnù, Anna Colombo, Cosimo de Filippis, Alessandro Martini, Niccolò Favaretto

**Affiliations:** 1 Neurosciences Department, Università degli Studi di Padova, Padua, Italy; 2 Section of Otorhinolaryngology-Head and Neck Surgery, Azienda Ospedale Università Padova, Via Giustiniani 2, 35128 Padua, PD Italy; 3 Section of Otorhinolaryngology-Head and Neck Surgery, Azienda ULSS 5 Polesana, Rovigo, Italy

**Keywords:** Hearing loss, Auditory perception, Brain involvement, Aging, Dementia, Cognitive impairment

## Abstract

Hearing loss in the elderly is a very common disease: it is estimated to affect up to a third of the population aged 65 years or more, and 50% of people over 75 years old. There is a growing amount of data concerning the association between hearing loss and cognitive decline. Various possible mechanisms at the basis of this association have been proposed, such as the “common cause hypothesis,” the “cascade hypothesis,” and the “cognitive load hypothesis.”

Critically reviewing the data is essential to highlight the features of the relationship between hearing loss and cognitive decline. Most of the hearing tests available should take into account that dementia or even just mild cognitive impairment (MCI) may lead to poor performance during examination. On the other hand, clinicians should also remember that tests used to assess cognitive function require an adequate hearing capacity.

In this article we propose to analyze current diagnostic tests, treatment options, auditory processing, and rehabilitation strategies for hearing loss in the elderly in order to facilitate the management of this handicap in this fragile population.

## Introduction

### Hearing loss and cognitive decline

Hearing loss in the elderly is a very common disease: it is estimated to affect up to a third of the population aged 65 years or more [1], and 50% of people over 75 years old [2]. Projections for the future predict that there will be 300 million elderly people with hearing loss by 2030, that will become 700 million by 2050 worldwide. Alongside this, there is a growing amount of data concerning the association between hearing loss and cognitive decline. Some authors suggest that there is a dramatically increased risk for incident dementia in people with hearing loss: nearly twice the risk compared to the normal hearing population for mild hearing loss, three times for moderate hearing loss and five times for severe hearing loss [3]. Additionally, various possible mechanisms at the basis of this association have been proposed. There are three mechanisms that have been considered to be the most important in the development of these conditions, with different impacts in different patients [4]. The first one is often called “common cause hypothesis” and claims that both central nervous and auditory systems suffer the neurodegenerative effects of age or other noxae, and therefore together proceed toward deterioration. The second one, called the “cascade hypothesis,” argues that hearing loss causes cognitive decline in a combined way: impoverished sensorial input, social isolation, and an increased depressive mood, directly caused by hearing loss, may concurrently worsen cognitive functions. The third hypothesis is referred to as the “cognitive load hypothesis,” which suggests that in patients with hearing loss the normal cognitive resources allocated for hearing, and in particular speech comprehension, become insufficient and therefore other resources must be redirected to maintain an adequate hearing performance. These changes may reduce the resources available for other cognitive tasks (such as working memory) and consequently accelerate cognitive decline.

Although the association between hearing loss and cognitive decline is solidly established, critically reviewing the data is key in highlighting the features of the relationship between the two conditions. In a recent meta-analysis [5], the authors show how often the only audiological measure to assess hearing loss is pure tone audiometry, the simple assessment of the response threshold for sounds at different frequencies, or even just the pure tone average, the mathematical average of the hearing threshold at 500, 1000, 2000, and sometimes 4000 Hz. These measures are very often insufficient, though necessary, to assess the actual hearing performance of the patient: a certain degree of hearing loss may be present, but the shape of the hearing loss (i.e., whether it affects all frequencies, or mainly high or low frequencies, etc.) and also other elements may affect how patients perform in real-life situations. For current studies design, other measures for assessing hearing performance are routinely used mainly to better characterize speech intelligibility in different hearing conditions.

Moreover, many previous studies have only assessed the presence of full-blown dementia in association with hearing loss, while nowadays there is growing interest in the stages that precede dementia, that are collectively called “mild cognitive impairment” (MCI). The main reason is that in this stage of cognitive decline, interventions aiming to slow its development seem to be more effective. Clinical tools that are commonly used to screen the presence of dementia are far less capable of detecting MCI: the mini-mental-state examination (MMSE), which has been the most widely used screening tool for cognitive impairment, is currently undergoing examination due to its very low sensitivity in detecting MCI [6].

Lastly, two important aspects must be taken into consideration. Firstly, nearly all the tests used to assess cognitive function require an adequate hearing capacity, otherwise cognitive impairment, especially in comprehension, may be overestimated; secondly, cognitive assessment tests and hearing examination require the patient’s attention and collaboration to complete the required tasks, which are often very difficult for an old patient. Therefore, in subsequent tasks, cognitive weariness may arise and be added to the overall stress of the clinical examination, consequently modifying the overall outcome of the tests.

### Diagnosis of hearing loss in the elderly in the audiology clinic

In adults and in the elderly the diagnosis of hearing loss is not particularly challenging, especially if the patient is cooperative. Common and widely used hearing assessment tools are pure tone audiometry and speech audiometry. First of all, patients are examined via otoscopy in order to exclude any visible obstacle or disease that may impair hearing.

Pure tone audiometry consists in a psycho-acoustic test in which patients, in a sound-proof booth, are asked to raise a hand or press a button when they hear a sound presented in earphones or with a free speaker in the booth (free-field pure tone audiometry). Each sound presented is a pure tone of a specific frequency and can be modulated in intensity level, measured in dB hearing level (HL), a unit of measurement which takes into account the different sensitivity of the ear for different frequencies and normalizes the value. Sound can be given in a single ear at a time, and if the response threshold of the two ears shows a consistent difference, masking may be necessary. Masking consists in providing a continuous sound in the better-hearing ear in order to avoid that a high intensity sound presented in the worse ear is heard with the contralateral ear (via bone conduction). Also, sound can be provided by a bone vibrator positioned behind the ear, to assess the bone conduction threshold, as opposed to the air conduction threshold provided by the earphone and free-field audiogram. Air conduction assesses the functioning of all the parts of the ear (external, middle and inner ear), whereas bone conduction bypasses the external and middle ear and directly stimulates the inner ear. Differences between the two thresholds may imply an underlying condition of the external or middle ear, such as otosclerosis, otitis media, or ossicular malformations.

Speech audiometry is used to assess the actual comprehension of speech: words instead of pure tones are presented to the patient, who must repeat them correctly. Different lists of words are presented at different intensity levels, and the percentage of correctly reported words is registered. Then, three intensity level thresholds are reported: the detection threshold, which represents the level at which the subject hears something, but he/she is not able to provide any correct word, the recognition threshold, which is when the patient identifies 50% of the correct answers, and the intelligibility threshold, when all the answers are correct. Since intelligibility and even recognition thresholds may sometimes not be reachable, another value considered is the word recognition score, which represents the maximum percentage of correct word identification reached by the patient. Speech audiometry is influenced far more by the cognitive status of the patient because it is a more difficult task than pure tone audiometry, and also by the patient's proficiency in the language used for the test.

In addition to these two traditional tests, in the last few years the International Matrix Sentence Test (IMST) has been implemented in clinical practice in many Audiology Clinics. Since both of the previous tests are performed in a silent booth, they do not accurately represent the real-life situation of the patient, because the acoustic environment of everyday tasks is far richer in noise and other competing signals. Speech-in-noise comprehension deterioration seems to be one of the first signals of hearing loss, especially in the elderly, and patients in the Audiology Clinic frequently complain of this symptom. The IMST test tries to reproduce these conditions in the clinic: the automated system generates 5-word sentences that are grammatically correct but semantically unpredictable and presents them to the patient together with a noise at different sound-to-noise ratios (SNR). Patients must repeat all the words that they can identify, and the system automatically changes SNR and presents another sentence. At the end of the exam, the test shows the minimum SNR value that allows the patient to correctly repeat at least 50% of words. Normal values differ between languages, and language proficiency greatly influences this exam as well.

Alongside this standard clinical evaluation, patients may require electrophysiological exams, such as auditory brainstem evoked potentials (ABR), or radiological imaging (CT, MRI), according to the characteristics of each case. ABR is an objective evaluation of the auditory pathway function from the auditory nerve to the mesencephalon. Despite the ABR not being used in routine clinical practice to evaluate elderly hearing loss, it may be useful in assessing the residual neurosensorial threshold in patients who do not fully cooperate. Aging reduces amplitudes of all principal ABR peaks, reducing the numbers and/or the synchrony of contributing units in the auditory nerve and cochlear nucleus [7]. What remains to be defined is the physiological mechanism of this phenomenon. In fact, studies with animal models have been made to investigate if the reduction of amplitude is due to a “peripheral” or “retrocochlear” involvement. Cai et al. [8] observed that age-related changes involve wave I and wave V amplitudes, with a significant overall increase in the I/V ratio due to a larger age-related wave I amplitude reduction, compared to wave V reduction. Moreover, regression analysis showed a strong correlation between IHC-SGN synapses number and wave I amplitude in the same animals, even though species and strain differences have been observed in evaluating the causes of decreased peripheral input in age-related hearing loss.

In the animal model, it seems that neural inhibition is progressively reduced with aging at the level of the midbrain. This phenomenon seems to explain the subsistence of the late waves of the ABR, while aging acts progressively on the loss of the peripheral synapsis. These electrophysiological mechanisms might be the explanation of the age-related progressive decrease in the temporal resolution and speech discrimination tasks even in the “normal hearing” elderly (see the paragraph “Evaluation of different domains of auditory processing”).

In addition and in relation to cognitive impairment, further specific tests and assessments may be necessary to evaluate different aspects of hearing and cognitive auditory processing.

### Treatment options

Generally speaking, auditory rehabilitation with hearing aids is the most common and effective treatment available in most cases. Surgical treatment is limited to specific diseases (otosclerosis, damaged eardrum) and in some cases to ones that can cause severe complications (chronic otitis media). Therefore, in some cases surgery might even worsen the hearing status, due to the need to remove the external or middle ear structures that contribute to the transmission of sound to the inner ear. Hearing aids are commonly suggested or prescribed by the physician when the hearing threshold significantly impairs the speech comprehension in everyday life. Hearing aid configuration and fitting is provided by the audiologist, who adjusts the device according to the patient’s audiogram and needs. Hearing aids have become more and more sophisticated and technologically advanced in the last few years, providing a great variety of possibilities in sound amplification, compression, and probably most of all in sound pre-processing: scene analysis to reduce noise, directional microphones, and connectivity accessories have greatly improved hearing aid effectiveness, but have also raised costs. In some countries hearing aid provision is partially or completely paid for by the health care system but in some others it is not, or not completely, potentially preventing access to these devices for lower income families. It is therefore important that the choice of a hearing device is adapted not only to the patient’s hearing status/necessities but also to the actual socio-economic conditions.

In case of severe to profound hearing loss, amplification by means of hearing aids may not be sufficient to guarantee a good auditory performance, making the cochlear implant the best choice for these patients. Cochlear implants were first developed in the 1960 s [9] and have become more and more effective in providing a serviceable hearing in cases of profound hearing loss. Due to technical aspects, hearing provided by a cochlear implant is substantially different from physiological hearing: the perception of rhythm (temporal perception) is similar to normal hearing and hearing aid users, but the perception of pitch (spectral perception) is far worse [10]. Speech is a highly redundant signal (i.e., a signal with auditory cues exceeding what is usually necessary to understand speech) and for this reason speech intelligibility is easily achievable with a cochlear implant. At first, cochlear implant surgery was reserved only for children with congenital deep hearing loss, but the potential improvement for adults and even the elderly has become more and more evident. Both adults and the elderly may significantly benefit from cochlear implant in terms of hearing outcome and quality of life [11]. Careful audiological counseling is mandatory before cochlear implant surgery, along with the support of a speech therapist: after the surgery and the activation of the device, the patient needs to undergo speech therapy in order to train the auditory system to interpret and decipher the stimuli provided by the cochlear implant. Also, cognitive evaluation is a fundamental part in counseling, since weak cognitive resources may interfere with speech training in cochlear implant users and patients with severe cognitive impairment may not benefit at all from a cochlear implant. Nonetheless, poor cognitive performance is not an absolute contraindication for the choice of the cochlear implant as a rehabilitation strategy, but it may affect the pre and post-surgical management.

### Beyond age-related hearing loss: auditory processing in the elderly

In some cases, patients who come to the Audiology Clinic may complain of specific hearing problems: difficulties in hearing in noisy environments, in hearing people that talk fast or even “hearing without understanding,” meaning that they can hear the sound itself, but not the information it carries. The pure tone hearing threshold in these patients often shows mild or moderate hearing loss, in contrast with a poor performance at the IMST or even at the speech audiometry. In these cases, rather than a sensory impairment, hearing difficulties are ascribable to an altered processing of the auditory information. Sound contains various types of information: where the sound comes from (spatial information), how the sound changes in time (temporal envelopment and temporal fine structure), and pitch and timbre contents (spectral information). All these characteristics are encoded by the hearing system and analyzed by the central nervous system in order to extract the information. Also, sound comes to the central nervous auditory regions through two ears, requiring neural integration of the two signals to avoid interaural interference.

All these functions may be altered in an aging brain, especially when a certain degree of cognitive decline is also present, in particular, when working memory is involved [12]. The evaluation of these aspects, together with a comprehensive analysis of cognitive decline, may lead to a better understanding of how cognitive decline and auditory processing are related and, possibly, to the development of specific rehabilitation strategies.

### Evaluation of different domains of auditory processing

Many tests that evaluate auditory processing have been developed over time, and each is specific for a particular auditory process.

One of the most studied functions, which seems to be related to impaired speech perception when altered [13], is temporal processing. Among these tests, the gap-in-noise test has been extensively studied and provides data relatable to speech acoustics: in this test subjects are asked to detect a brief interruption in continuous noise. Normal young subjects are capable of detecting gaps up to 3–4 ms [13, 14], while the elderly are only capable of detecting longer gaps. The ability to detect brief changes in sound over time, when applied to speech, becomes important in order to detect brief sounds such as consonants, especially stop consonants. The gap-in-noise test is simple and repeatable and requires a moderate degree of collaboration and attention. This test, however, may be imprecise due to the non-verbal nature of the stimulus. For this reason, some authors [15] have conducted tests with speech and non-speech stimuli and have confirmed that in both these conditions the detectable gap was longer for the elderly compared to the control group.

Another auditory processing function is the ability to distinguish sounds of different frequencies and frequency composition. This ability is called spectral resolution. One of the tests that tries to highlight this function is the spectral ripple test, in which a sound made up of a broad band of frequencies is presented to the patients. In this stimulus the frequency is amplitude modulated. In other words, the relative intensity increases from lower to higher frequencies, reaches a peak, and then decreases, describing various crests, called “ripples.” The number of ripples can be varied in order to obtain sounds with closer or farther crest tips. This particular sound is then presented to the listener, who is asked to discriminate it from a broad band noise or from another spectrally rippled stimulus with a different number of ripples. The ability to distinguish between stimuli with a high and similar number of ripples per octave reflects a good spectral discrimination capability. An association has been reported between spectral ripple test performance and hearing in noisy environments, especially in cochlear implant users [16]. More recently, to avoid possible loudness cues caused by crest tips that may lead to the identification of the sounds regardless of their spectral content, a modified spectral ripple test has been developed [17]: the spectral temporally modulated ripple test (SMRT) also uses amplitude-modulated broad band sounds, but the modulation varies over time, shifting the crests along the frequency range of the sound while it is played. In this test, specific loudness cues cannot be identified because they are not static over time, but the spectral distance between crests is maintained, thus allowing the listener to spectrally discriminate the stimuli. SMRT test performance has been reported in relation to speech hearing in noise [17] and could potentially reveal useful information for a more precise device fitting for hearing aid users.

Lastly, another important aspect of auditory processing is binaural integration. In the presence of inadequate central auditory functioning, the signals coming from both ears may interfere with each other, resulting in a deterioration in auditory perception. To evaluate this aspect, dichotic listening tests are usually implemented. In these tests, the patient is synchronously presented with two different stimuli, one in each ear, and is asked to repeat them. The stimuli used may be phonemes, words, and sentences. A similar test to evaluate binaural integration is the staggered spondaic word test, in which two different two-syllable words are presented one in the right ear and one in the left ear not entirely synchronous: the second syllable of the first word and the first syllable of the second word overlap, thus allowing central cognitive integration in understanding the two words, using the syllables that are not overlapped as semantic cues. When these tests are altered, typically patients show right-ear dominance: the stimulus that is correctly identified is more frequently the one presented in the right ear, which occurs because the pathway to the auditory cortex is more direct for the right ear than for the left. Like the previous test, an altered binaural integration may imply different rehabilitation strategies.

### Possible implications of auditory processing in aural rehabilitation

Considering the complexity of sound and the high technological development in hearing aids and cochlear implants, highlighting specific auditory central functioning of patients may lead to more tailored and efficient methods of aural rehabilitation, and consequent improvement in hearing and daily life.

A patient showing binaural integration impairment, for example, may not benefit from a bilateral hearing aid. Conversely, the patient may get a better advantage from using only a single hearing aid (preferably in the right ear), if the hearing threshold is similar between the two ears. Reduction in spectral resolution may direct the audiologist to specific compression strategies for sounds, even if compression may not work with a perfect spectral resolution, to the point in which traditional analog hearing aids may result similar in hearing outcome than more modern digital ones. Lastly, temporal processing ability decay is probably the most challenging central auditory condition to treat. To properly balance this impairment, a combined cognitive and audiological rehabilitation program could be useful, to implement and train different cognitive strategies capable of reestablishing a hearing that is good enough to sustain social activities in daily life.

In general, reduced compliance to hearing aids use is frequently considered a sign of poor cognitive performance or psychological disturbances, but we should consider that hearing difficulties or inadequate hearing aids’ fitting may lead to frustration for the patient. In certain cases, the discomfort can be reduced by a tailored fitting based on an in-depth audiological evaluation, thus facilitating the overall management of fragile patients. In general, a good auditory outcome is not impaired by the age of the patient [18] or by how long the patient has had auditory deprivation [19], but clinicians and families should be aware that the best results might require longer than younger patients. Moreover, new hearing aids and cochlear implants enable Bluetooth® connection with smartphones and microphones, thus improving hearing performance and remote communication, which may be particularly advisable in a time in which fragile patients should be protected by all means [20].
